# The Vassar Girls

**Published:** 1887-07

**Authors:** 


### THE VASSAR GIRLS.

The report of the Treasurer of this famous female college shows that its inmates have consumed, during the last academic year, among other things 84,000 pounds of fresh meats, 8,000 pounds of smoked meats, nearly 5,000 pounds of turkeys, over 4,000 pounds of chickens, nearly 4,000 pounds of fish, 32,000 clams, 141 gallons of oysters, 230 barrels of flour, 14,000 pounds of butter, 95,000 quarts of milk, 12,000 pounds of sugar, 30,000 oranges and lemons, 10,000 bananas, over 1,000 bushels of potatoes, and 100,000 buckwheat cakes It is to be hoped that this burden, or perhaps we should say *privilege*, will be, hereafter, more evenly distributed among the young men of the country.

